# Trojan Microparticles for Drug Delivery

**DOI:** 10.3390/pharmaceutics4010001

**Published:** 2012-01-06

**Authors:** Nicolas Anton, Anshuman Jakhmola, Thierry F. Vandamme

**Affiliations:** Laboratoire de Conception et d’Applications de Molécules Bioactives, Faculty of Pharmacy, University of Strasbourg, CNRS 7199, 74 route du Rhin, 67400 Illkirch, France

**Keywords:** pharmaceutical nanotechnologies, microparticles, microspheres, vectors, solubility, permeability, dosage form, administration routes, drug targeting, release, nanoemulsion, trojan

## Abstract

During the last decade, the US Food and Drug Administration (FDA) have regulated a wide range of products, (foods, cosmetics, drugs, devices, veterinary, and tobacco) which may utilize micro and nanotechnology or contain nanomaterials. Nanotechnology allows scientists to create, explore, and manipulate materials in nano-regime. Such materials have chemical, physical, and biological properties that are quite different from their bulk counterparts. For pharmaceutical applications and in order to improve their administration (oral, pulmonary and dermal), the nanocarriers can be spread into microparticles. These supramolecular associations can also modulate the kinetic releases of drugs entrapped in the nanoparticles. Different strategies to produce these hybrid particles and to optimize the release kinetics of encapsulated drugs are discussed in this review.

## 1. Introduction

Nowadays, nanotechnology is a multidisciplinary scientific field that is currently undergoing a large technological development. Nano-enhanced medicines are opening new doors for therapeutic delivery and targeting. These new technological advancements are finding new solutions for using efficiently (as for conventional formulations) a number of bioactive molecules having a poor bioavailability, insolubility or drug instability. This results in an unprecedented multidisciplinary convergence of scientists dedicated to the study of a world so small. Nanotechnology is rapidly becoming an interdisciplinary field since biologists, chemists, physicists and engineers are all involved in the study of the properties of substances at the nanoscale regime. The main reason of interest in this field is because the nanoscale is the first point at which molecules can be assembled.

Amongst the different industrial potentials, nanotechnology also has a high impact on the medical industry. With this kind of technology, patients can be administrated with fluids containing nanorobots or nanoprobes programmed to attack and reconstruct the molecular structure of cancer cells and viruses. There is even speculation that nanorobots could slow or reverse the aging process, and life expectancy could increase significantly. Nanorobots could also be programmed to perform delicate surgeries; such nanosurgeons could work at a level which can be a thousand times more precise than the sharpest scalpel [1].

Today, the main application of nanotechnology lies in the administration of nanoparticles (NPs) containing bioactive molecules as well as contrasting agent, for biomedical applications. NPs for nanomedicine are made from many different materials such as polymeric, inorganic, lipid, but all with the same purpose, which is the controlled delivery of drug or contrasting agent for diagnosis. In comparison with conventional formulations (e.g., macro-formulations like tablets, or micro-formulations like microspheres, microcapsules *etc*.), NPs present many advantages. The nanoscale allows better penetration of the colloids through biological tissues, through biological barriers and also through cellular membranes. It therefore follows that the efficiency in bringing the active molecules towards the administration sites is significantly increased; thus, for instance, improving drug absorption. The classical administration routes for the NPs are oral, pulmonary, dermal, rectal and injectable. However, still today, the main challenge of nanomedicines lies in the efficient administration of these NPs. The difficulty is the manipulation of NPs in order to bring them to the absorption sites, e.g., pulmonary alveoli, intestinal barriers, mucous membranes, *etc*. This is particularly true in the case of pulmonary administration. On the one hand, the size range of nanoparticles is too small to insure their retention in the deep lung: actually, NPs can be inhaled, but they are totally expelled during exhalation. Ideal particle sizes for the pulmonary alveoli administration are between 2 and 5 µm, corresponding to the typical size of microparticles. On the other hand, while the best systems for targeting the pulmonary alveoli are such microparticles, the latter are too big to diffuse and allow an efficient administration of the encapsulated drug as NPs can. The result is a real dilemma. Actually, a solution has been found by combining micro and nanoparticles, namely the encapsulation of nanoparticles in microparticles. The microparticles are brought onto the alveoli, and specifically release the NPs, which can diffuse through the barriers. Such multiscale nano-in-microparticles systems are called *Trojan particles*. Many different other potential applications of these Trojan systems are reported, and tailored in function for the administration routes, e.g., oral, dermal, intraocular. In the case of pulmonary, Trojan particles overcome an important technical challenge that is to bring and concentrate the NPs onto the specific absorption sites above-mentioned. In the present article, we propose a detailed review of the technologies inherent to the Trojan microparticles, by presenting in the first part, the technical details of their fabrication, processes and formulation methods, and, in the second part, by reviewing the potential of Trojan microparticles in terms of biopharmaceutical aspects and applications.

## 2. Fabrication, Physical Principles and Experimental Procedures

In the context presented above, it is clear that this type of multiscale microsystem requires a multistep formulation procedure which involves two major steps. These steps are (a) fabrication of the nanoparticulate suspension, and (b) their integration with the microparticles.

### 2.1. Nanoparticles

In the present review, we will only discuss nanoparticles as an entity to be encapsulated for the fabrication of the Trojan particles, rather going through their synthesis aspect. The encapsulation of drugs into nanoparticles has been widely reported in literature [2,3,4,5,6]. Nanoparticles are generally defined as rigid colloidal particles having sizes from 1 nm to 1 μm. They are fabricated from macromolecular and/or molecular assemblies, polymers or lipids, in which the active principle is either dissolved, entrapped, encapsulated, adsorbed or attached to the external interface. The main advantage of nanoparticles as compared to other colloidal drug delivery systems (liposomes, niosomes, *etc*.) lies in their unique structural properties and stability. This point is of fundamental interest in order to maintain their size and morphology when they undergo the microencapsulation processes during the fabrication of Trojan microparticles. Nanoparticles are generally divided into two main families: nanospheres (which exhibit a homogeneous matricial structure) and nanocapsules (which have typical core-shell type morphology). The formulation processes as well as the nanoparticle structure are tailored according to the requirements in terms of drug encapsulation and drug delivery (biocompatibility of the polymer, physicochemical properties and solubility of the drug, therapeutic goals).

The unique features of nanoparticles depend on their shape and size, chemical nature and/or surface functionalization allowing their diffusion into the tissues or organs at the administration site. NPs penetrate the cell membranes and are able to enter the cells located in these tissues [7]. Due to this, the delivery of bioactive molecule is not only enhanced in efficiency, but also such nanocarrier-based technologies allows the protection of the fragile encapsulated molecules, or they may protect living tissues from cytotoxic molecules, until they reach their target [6]. To summarize, the two fundamental points inherent to the nanoparticulate systems are (i) their nanometric size, and (ii) the encapsulation of active pharmaceutical ingredient (API). These two points should be taken into account, and thoroughly controlled when the microencapsulation of nanoparticles is designed for the fabrication of Trojan particles.

Accordingly, the microencapsulation processes should, neither destroy the nanoparticle morphology, nor induce premature drug release (during processing) [8]. Besides, the Trojan fabrication processes have to be designed according to the chemical nature and physico-chemical properties of the nanoparticles suspension. These processes are discussed below; in the literature the most adopted technique to design such particles is the spray-drying process, which is quite compatible with such specifications.

Another essential point related to the therapeutic goals and bioactive delivery lies in the API leakage over time, which is linked with nanoparticles’ loading capabilities, and their release profiles. Actually, one interesting point of the Trojan particles is the modification of the release profiles, which reduces the burst effect and slows the kinetics [8], finally resulting in improving and optimization of the API delivery.

### 2.2. Microparticles

The second experimental challenge lies in the formulation of microparticles with the constraints presented above. The spray drying process with few modifications is most often used for the fabrication of Trojan particles (detailed below). Some modified spray drying methods using supercritical CO_2_ as antisolvent medium are also reported and are particularly efficient for the formulation of Trojan particles (e.g., in [9,10]).

A spray dryer apparatus is generally used to transform liquid substances into powder materials quite rapidly and efficiently. This drying process is performed by atomization, which involves a very short drying time, thus enabling the drying of temperature-sensitive materials without degradation [11,12]. The spray drying process is largely used to improve product conservation in dried solid form. Moreover, as illustrated in the current review, with the development of technologies for encapsulating active compounds, emulsions, nanoparticles (or nanostructured materials), used in pharmaceutics, cosmetics and functional food preparation, this method has also been largely studied and used for encapsulation purposes. The powder generated is generally in the form of heterogeneous and amorphous microparticles, which can exhibit various structures, for example matricial (*i.e.*, microspheres [13]) or hollowed morphologies. Particle structure, size and morphology, depends on various parameters, from physicochemical properties of the materials to the thermodynamic parameters and phenomena related to the two-phase flow in spray dryers.

Actually, owing to the complexity of the phenomena involved, and due to a large number of processing and formulation variables, the physical description of the process and its effects on the resulting particles size and morphology is not well understood [11]. To summarize, the structural and morphological properties of the microparticles formed by spray-drying is a function of the feed solution concentration [14] as well as the solubility of the excipients [15,16,17]. Likewise, other physico-chemical aspects, like precipitation kinetics and crystallization have also shown significant impact on (and/or can be affected by) the evaporation rate [18,19] and the drying temperature, which have a direct effect on the resulting particle morphology [20,21].

On the other hand, Tsapis *et al.* [22] emphasized the importance of another parameter, the Peclet number (Pe), in understanding the formation process, morphology and density of the particles. The Peclet number is a dimensionless mass transport number that characterizes the relative importance of diffusion and convection [23]. Pe actually gives an idea as to whether the microparticle structure will be a matricial, or rather hollow like “empty microcapsule”. This number is given by the ratio between two characteristic “times” which are critical for the drying process: the first one, *τ_d_*, is the time spent by droplet in drying, and the second one, *R^2^/D*, corresponds to the time of diffusion of a solute (molecule or nanoparticles), to move from the external interface to the center of droplet (where R is the droplet radius, and D the diffusion coefficient of the solute or nanoparticle).



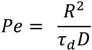
(1)


*Pe* appears to be closely linked to the processing parameters as well as to the chemical nature and mobility of the solutes. As a non-exhaustive list, this *Pe* is also influenced by the drying parameters like inlet temperature, pressure, gas flux (playing on *τ_d_*), as well as by the pressure of the spraying gas, viscosity, chemical nature of the solute, intermolecular interactions, concentration of the solute, factors related to the diffusion coefficients and to the droplet radius *etc*. On the other hand, Taspis *et al.* [24] and Marty and Tsapis [25] showed that the morphology of the particles could also depend on the mechanical properties of the material forming the wall, as well as on the colloidal interactions. This is studied through a parameter called buckling threshold, which is the time for which the surface of the droplets suddenly buckle like elastic shells. However, we chose here to focus the discussion below around the Peclet number, which allows describing and understanding, under a clear point of view, the relationship between the nature of the materials, drying process, and particle morphologies.

**Figure 1:**
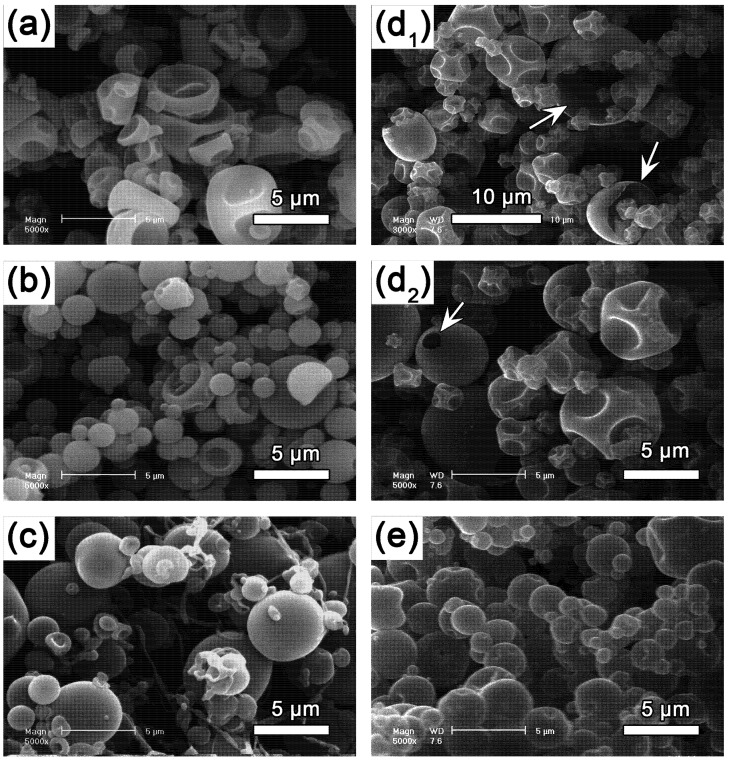
SEM pictures of microparticles of different materials and/or spray dried under different conditions. All powders, excepted the one shown in (**b**), are spray dried with the Büchi Mini Spray Drier B-290, in exactly the same operational conditions (inlet temperature: 150 °C, solution feed rate: 10 mL·min^−1^, aspiration rate: 100%), whereas sample (**b**) was obtained using a different apparatus, the Büchi Nano Spray Drier B-90 (with inlet temperature fixed at 100 °C feed rates at 10 mL·h^−1^, the drying gas flow rate equal to 100 L·min^−1^, and the spray mesh used in this study was the membrane with 4.0 μm sized holes). (**a**) Gum Arabic at 10 wt.% in water (B-290). (**b**) Gum Arabic at 10 wt.% in water (B-90). (**c**) Polyvinyl Alcohol at 15 wt.% in water. (**d**_1_) and (**d**_2_) Hydroxypropyl *β*-cyclodextrins (Kleptose^®^ HPB from Roquette, France) at 15 wt.% in water; arrows highlight particular structures due to high *Pe* discussed in the text. (**e**) Hydroxypropyl *β*-cyclodextrins (Kleptose^®^ HPB) at 15 wt.% in water, encapsulating oil-in-water nano-emulsions (*d_h_* = 85 nm) at a weight ratio of 1:4 (emulsion:polymer).

Figure 1 presents SEM micrographs of microparticles spray dried in different conditions and materials. These illustrations show the influence of such macroscopic parameters on the Peclet number. Figure 1 (a,b) present microparticles made from the solution of gum Arabic at 10wt% in water, but spray dried with different apparatus, like the Büchi Mini Spray Drier B-290 [26] and the Büchi Nano Spray Drier B-90 [27], respectively. The spray drying technologies in both the cases are significantly different from each other, and as a result, particles of different size range are obtained. The particle size distribution for both the cases are shown in Figure 1, for Figure 1(a): (2.17 ± 0.66) μm [26] and for Figure 1(b): (0.55 ± 0.27) μm [27]. Similarly, the shape of the particles is also influenced, appearing as “deflated balls” in the first case, and more spherical in the second case: this means that *Pe* is different in both of these cases, and it is higher with the B-290 apparatus. So far as the spray dried solutions are strictly the same, this is an example of the influence of the droplet radius *R* on *Pe* (see Equation 1). It is however to be noted that the decrease of R can also result in the decrease in the value of *𝜏_d_* (which goes to increase *Pe*), as was observed in Figure 1(b) where these two phenomena resulted in reduction of Peclet number.

Figure 1(c), corresponds to 15 wt% of PVA aqueous solution spray dried with the Büchi Mini Spray Drier B-290. Here the particles morphologies corresponded to a *Pe* value lower than the one observed for gum Arabic in Figure 1(a) (in similar experimental conditions). This can be explained by the decrease of the mean particle size, measured as (1.39 ± 0.53) μm for PVA, which was due to the decrease of the surface tension at the air/water interface. Measurements of the average air/water surface tension during the first 30 seconds after the formation of drop (short times mimicking the conditions during the spray drying process), revealed that the values for gum Arabic and PVA are significantly different from each other 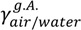 = (71.35 ± 0.01) mN·m^−1^ and 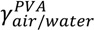 = (42.67 ± 0.10) mN·m^−1^. This result also explains the presence of “wire-like” objects formed during the droplet formation, which was due to an interfacial phenomenon. Here the surface tension measurements were performed using a drop tensiometer (Teclis, Longessaigne, France).

Another example is the spray-drying of hydroxypropyl *β*-cyclodextrins in Figure 1(d_1_) and (d_2_), performed at similar operating conditions as in Figure 1(a) and (c). The micrograph clearly shows that critical structures were formed due to very high Peclet numbers, indicated by the white arrows on the figures, hollow structures with a thin polymer shell, either opened (arrows) or closed (deflated ball-like) could be clearly seen in these microparticles. Compared to gum arabic as in (a) or PVA in (c), particles seem even more porous suggesting that the *Pe* number might be larger. Now, when a suspension of oily nano-droplets were added to the solutions spray dried in (d), the microparticle morphology as well as size changed drastically, presented in Figure 1(e). The particles appeared more spherical, probably correlated to a modification of the elastic properties of the particles. The size measured was (1.73 ± 0.56) μm (roughly similar to (d)), which could be due to the increase in *𝜏_d_* since the concentration of solutes in the droplets was consequently increased.

**Figure 2:**
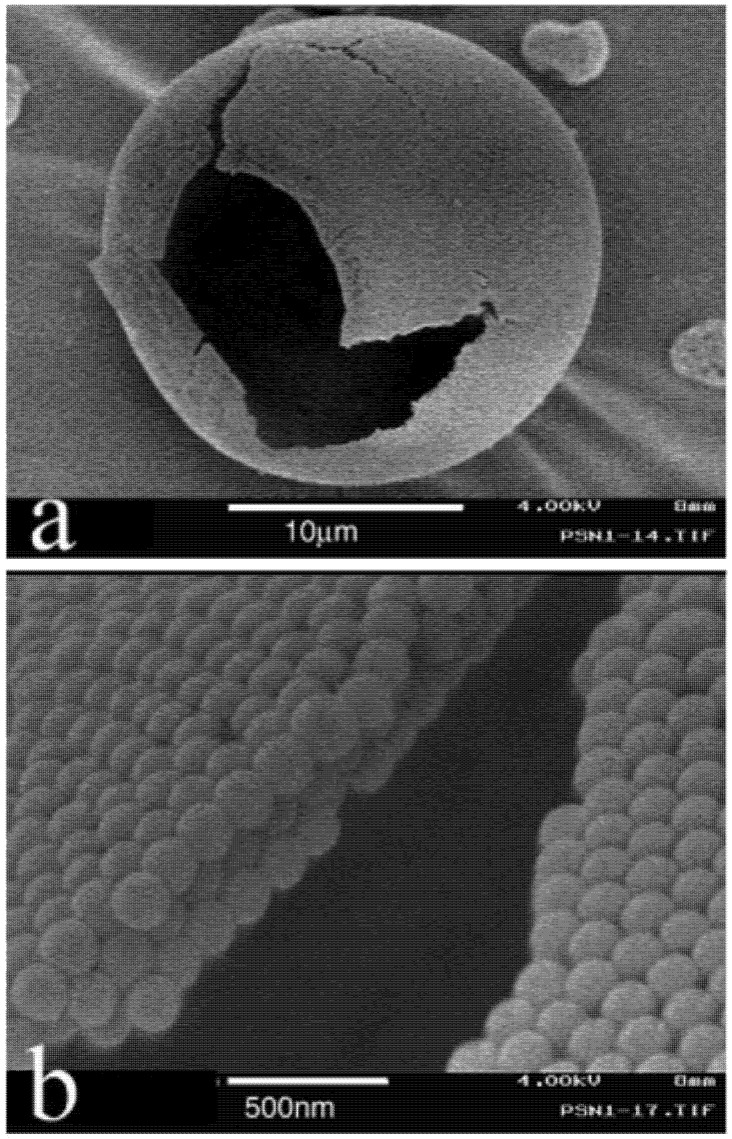
SEM pictures of (**a**) a typical hollow microparticle obtained by spray drying a solution of polystyrene nanoparticles (170 nm), and (**b**) magnified view of the particle surface in (a). Reproduced with permission from National Academy of Sciences, USA [22].

**Figure 3:**
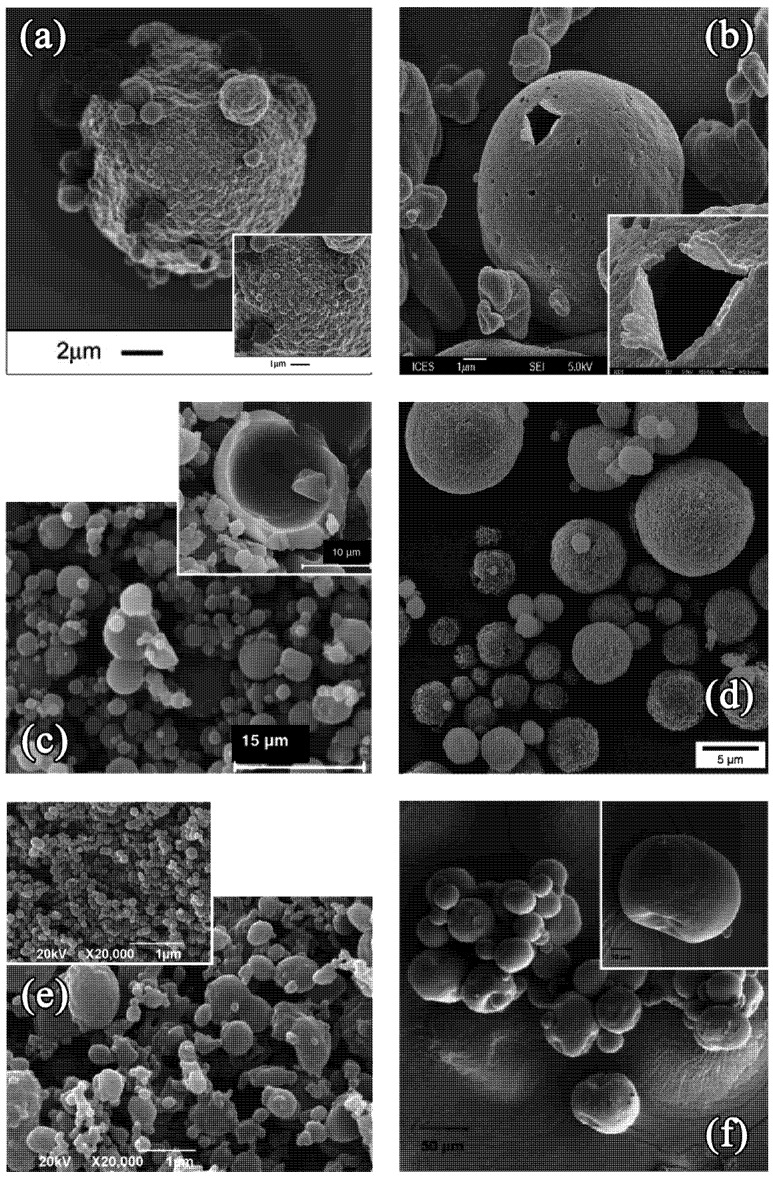
SEM pictures of a representative panel of Trojan microparticles reported in literature. (**a**) Reproduced with permission from Elsevier [7]: spray dried (Büchi B-191) microparticles prepared with DPPC, HA and PLGA nanoparticles; *Inset:* Close-up on Trojan particle surface: one can distinguish nanoparticles. (**b**) Reproduced with permission from Elsevier [42]: spray dried (Büchi B-290) microparticles prepared with DPPC and PMMA-MeOPEGMa nanoparticles. (**c**) Reproduced with permission from Elsevier [43]: spray dried (Büchi B-290) microparticles prepared with mannitol and chitosan/TPP nanoparticles. (**d**) Reproduced with permission from Elsevier [44]: spray dried (Büchi B-191) microparticles prepared with colloidal silicon dioxide as drying auxiliary and oily-core PCL nanocapsules. (**e**) Reproduced with permission from Elsevier [10]: spray-dried microparticles of PLLA in organic solvent (dichloromethane) in supercritical CO_2_, encapsulating puerarin nanoparticles also generated through this supercritical CO_2_ micronization process; *Inset:* Close-up on the puerarin NP after micronization. (f) Reproduced with permission from Elsevier [8]: PLGA microshperes obtained by a nonaqueous s/o/o/o [45,46] solid dispersion technique, encapsulating dexamethasone phosphate prepared with a supercritical CO_2_ nanoprecipitation process.

To summarize, we have seen that many different factors (operating parameters, formulation variables, nature of chemical) can have a significant impact on the Peclet number and particles morphologies. Accordingly, the molecular weight of solutes also have a significant impact on *D* and thus on the increase of *Pe*. Numerous examples are reported in literature with different morphologies, like hollowed, wrinkled, or dimpled particles (as also shown in Figure 1) for spray dried proteins [19,28,29,30,31,32,33], peptides [34,35], or polymers particles [17,36,37,38,39,40,41]. In this context, the spray drying of droplets containing nanoparticles can be considered as an extreme case when, compared to single molecules or macromolecules, or to the droplet surface, NPs can also be considered as immobile during the drying process. Accordingly, the diffusion coefficient is lowered and the *Pe* value is increased. The literature reports some examples of solutions composed of nanoparticles which are spray dried, this is reported in Figure 2 [22]. The results show hollow or “large porous” microparticles in which the cohesion between the NPs is due to weak forces between the interparticles. However, even if the nanoparticles induce an extreme reduction in *D*, the Peclet number does not become too high, as seen in Figure 1(e) in which a suspension of nano-emulsion mixed with a polymeric wall material (WM) was spray dried, details of which are given in [26,27]. The decrease of the diffusion coefficient *D* is counterbalanced by a decrease of the size and/or *𝜏_d_*, or even could be simply inhibited by the presence of polymers serving as wall materials. There are various examples in the literature describing the formulation of Trojan particles, which can either be formulated with high Peclet number (hollowed, wrinkled, or dimpled, e.g., as illustrated in Figure 2) or with a spherical morphology (as presented in Figure 1(e)). These results not only depend on the operating parameters discussed above, but also on the presence, concentration, and mechanical properties of wall materials (e.g., polymers) solubilized in the spray dried solutions. Figure 3 corresponds to SEM micrographs of Trojan microparticles obtained via different experimental approaches. The three first examples presented in Figure 3(a–c), respectively reproduced from [7,42,43], show multiscale assembly with polymeric nanoparticles spray dried in an aqueous media. All these examples produced high-*Pe* microparticles morphologies, with a shell thickness as a function of the wall material content. In Figure 3(a), poly(D,L-lactide-co-glycolide) (PLGA) nanoparticles were mixed with an aqueous solution of hyaluronic acid (HA) which were then spray dried. The authors observed a clear relationship between increase in *Pe* value with a decrease in WM-to-nanoparticle weight ratio (*i.e.*, an increase of the relative wall material content). This factor was fixed to 2:1 in Figure 3(a). The following micrograph, Figure 3(b), is of a similar system like that presented in Figure 2. This micrograph is of a dried suspension of NPs (without using any wall material).

As expected, few microparticles formed with the assembled nanoparticles (poly-methyl methacrylatemethoxy(polyethylene glycol)methacrylate (PMMA-MeOPEGMa) polymer nanoparticle) were hollow with very thin shell (even reaching a thickness about one nanoparticle), however these are extreme cases with high *Pe*. In the case where small molecular weight wall materials were used (as illustrated in Figure 3(c) with mannitol) for encapsulating chitosan/tripolyphosphate (TPP) nanoparticles (also where WM-to-nanoparticle weight ratio is fixed at 9:1), the diffusion coefficient of the solutes (WM and NPs) was found to be increased, resulting in decrease of *Pe* value. This resulted in spherical shapes, however bigger particles still appeared to be hollow, with a shell thickness of about 4–5 μm. Replacing WM by inorganic nanoparticles, resulted in highly geometric microparticle morphologies. One example is given in Figure 3(d), where spherical microparticles encapsulating oily-core poly(epsilon-caprolactone) nanocapsules (themselves encapsulating medium chain triglycerides), were spray dried using colloidal silicon dioxide as drying auxiliary. In contrary to the above examples, the particle morphology appeared quite homogeneous, spherical and seems to correspond to low values of *Pe*. This particular technology is described in detail in [47], where homogeneous particles with regular structures were obtained. The example shown in Figure 3(e) presents Trojan particles made by using the spray-drying technology in supercritical CO_2_ (as discussed above). The NPs obtained were dispersed in another organic phase (dichloromethane) containing a polymer (PLLA), and this mixture was once again spray-dried in CO_2_ in supercritical conditions. The last example of Figure 3(f) shows PLGA microspheres encapsulating drug nanoparticles, fabricated by the precipitation of PLGA by a specific mixing between polymer solvent and antisolvent.

**Figure 4:**
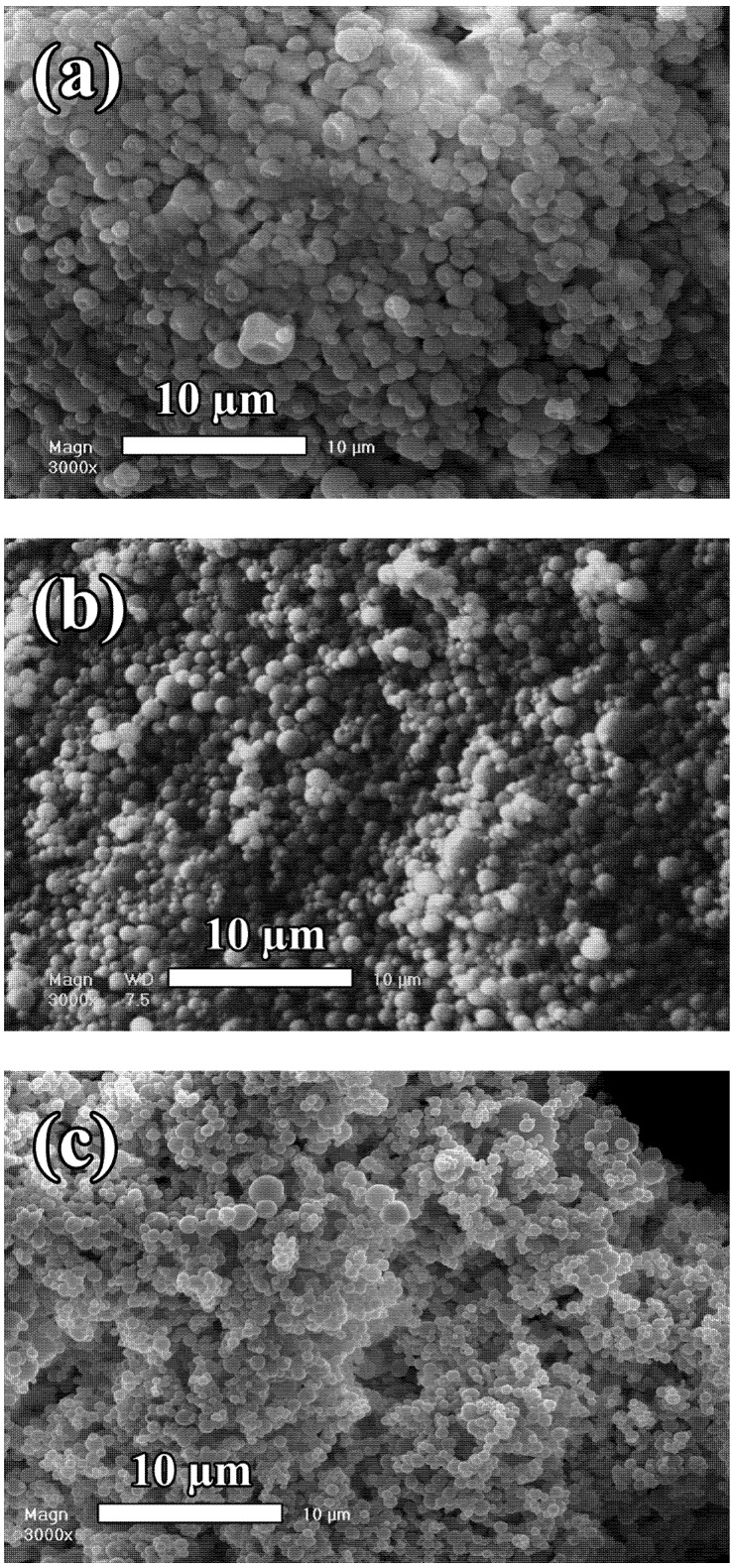
Trojan microparticles encapsulating nano-emulsions. (**a**) Wall materials: gum Arabic, Spray drier: Büchi Mini Spray Drier B-290. (**b**) Wall materials: gum Arabic, Spray drier: Büchi Nano Spray Drier B-90. (**c**) Wall materials: whey protein, Spray drier: Büchi Mini Spray Drier B-290. ((a) and (c) from [26], and (b) Reproduced with permission from Elsevier [27]).

Comparing the different experimental techniques reported here, the Trojan particles fabricated exhibit very similar size distributions, between one and ten micrometers. In view of the literature reviewed here, it seems that the size distribution of the Trojan microparticles generated are dependent on several factors, like the relative proportion between wall materials and NPs, the chemical nature of the materials used, the surface active properties of the NPs [26], and the apparatus used. In this way, we have recently reported [27] the possibility to generated submicronic Trojan particles encapsulating nano-emulsions by using the Büchi Nano Spray Drier B-90. This is illustrated in Figure 4 which presents Trojan particles encapsulating nano-emulsions using different wall materials like gum Arabic and whey protein, by using spray-drying apparatus: Büchi Mini Spray Drier B-290 and Büchi Nano Spray Drier B-90. In all these examples, exactly the same nano-emulsion was encapsulated in the different WM or using different spray drier apparatus. As discussed above with Figure 1, the drying technique strongly influences the *Pe* value as well as the size distribution of the particles, and it can be clearly seen that this behavior remains true even in the presence of nanoparticulate systems like lipid nanodroplets. Figure 4(a,b) presents the same nano-emulsion plus gum Arabic at identical concentrations, spray dried with the Büchi B-290 and B-90. The results clearly illustrate their influence in terms of size and Peclet number. The nature of the wall materials is also an important factor, which affects the microparticles’ morphology as illustrated, for instance with whey protein in Figure 4(c) compared to gum Arabic in Figure 4(a).

### 2.3. Impact on the Drug Delivery and Release Kinetics

**Figure 5:**
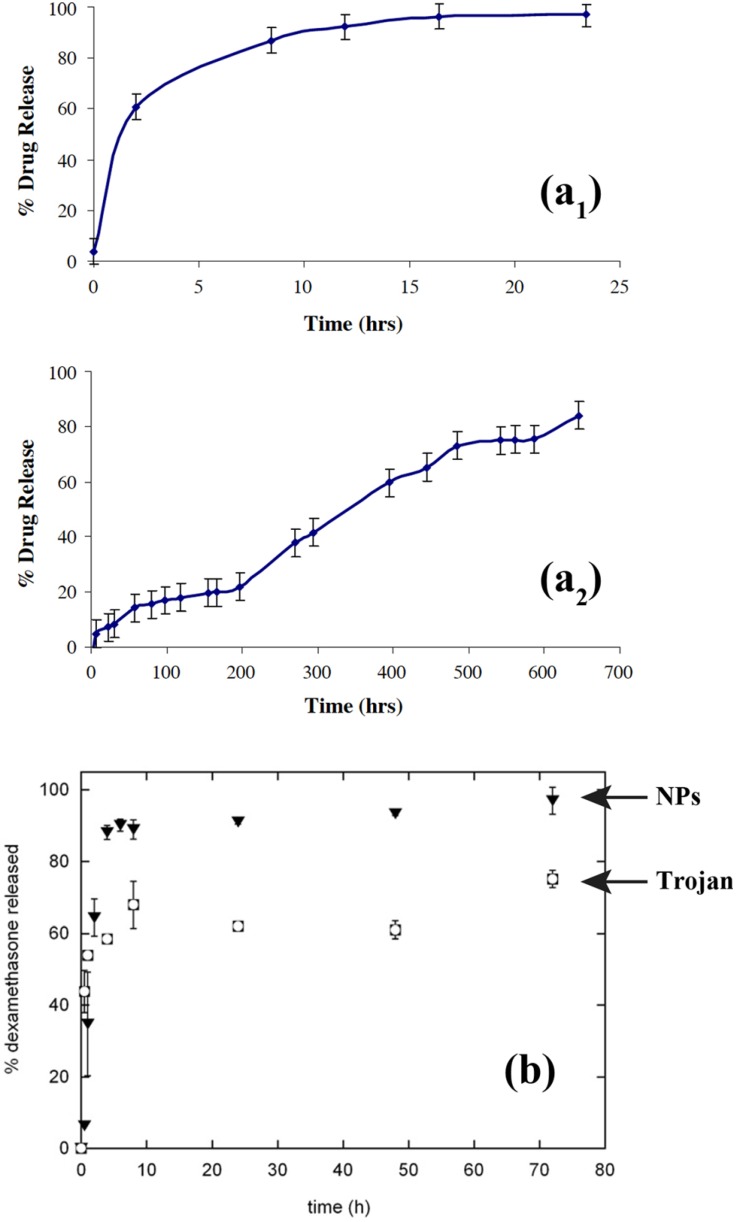
(**a**) Reproduced with permission from Elsevier [8]: (**a**_1_) *in vitro* release profile of dexamethasone phosphate from polylactidecoglycolide microencapsulated drug microparticles, and (**a**_2_) *in vitro* release profile of dexamethasone phosphate from Trojan microparticles: polyactide-co-glycolide microencapsulated drug nanoparticles. (**b**) Reproduced with permission from Elsevier [7]: *in vitro* release profiles of dexamethasone from nanoparticles (NPs) and from Trojan particles in HEPES buffer under sink conditions.

Although Trojan particles offer new possibilities for increasing the drug loading as well as its diffusion through living tissues, another important point lies in the drug release kinetics which can be modified using this technology. Most of the reports dealing with Trojan particles for drug delivery open a new possibility of a decrease in the burst release of the drug, and even in reduction of the release kinetics. Details of these can be found, in [7,8], illustrated in Figure 5(a,b), respectively. The first one (a), corresponds to Trojan microparticles as shown above in the SEM micrograph in Figure 3(f) made up of drug (dexamethasone) nanoparticles encapsulated in PLGA microparticles. Figure 5(a1) shows the drug release profile when the drug is encapsulated in microparticles, while Figure 5(a2) corresponds to Trojan particles. Here it can be seen that not only the burst effect was inhibited, but the drug release kinetics was also reduced. In this example, the resulting release profile was close to a zero-order process, which was likely governed by the polymer erosion. In other cases, like that presented in Figure 5(b), the drug was first encapsulated in polymeric nanoparticles (PLGA), followed by encapsulation in another polymeric matrix (hyaluronic acid). Here also, the burst effect was reduced with the Trojan system (open squares) compared to the drug release from the nanoparticles (filled triangles). However, the shapes of the two curves remained comparable *i.e.*, they could be driven by the drug diffusion at non-constant activity. This latter behavior is widely reported in literature and simply depends on the physicochemical properties of the Trojan system, like chemical nature of polymers and drug [42,48,49,50]. Actually, this phenomenon is still unexplained and there is no hypothesis to explain such a phenomenon. Moreover, establishing a general rule for the drug release from Trojan particles is not possible, as (i) all the Trojan particles are potentially different, the drug encapsulated can show different physicochemical properties, and (ii) the processing (e.g., spray drying conditions) as well as the formulation (e.g., drug concentration) parameters can play a significant role in the kinetics profiles. Overall, the drug leakage from Trojan particles is a result of combination of many factors like the drug release from the nanoparticles and microparticles matrix, along with the nanoparticles leakage from the Trojan matrix.

## 3. Biopharmaceutical Applications

In order to optimize the pharmacological activity and the therapeutic action, each drug has to be formulated in a particular dosage form with excipients improving the therapeutic effectiveness of the drug. Indeed, the therapeutic action of extra-vascular (pulmonary, oral, dermal, nasal and rectal) administrations is related to drug bioavailability. This is a quantitative and kinetics concept measuring the rate and the amount of a drug to penetrate the different membrane barriers allowing it to reach the target (organs, tissues, receptors). Therefore, the final goal of a biopharmaceutical development is to design a dosage form which can allow drug release “on demand” and to be able to reach specific targets. This approach also contributes to improve the socio-economic and industrial aspects. The biopharmaceutical developments of accelerated, sustained or prolonged drug release from a dosage form can illustrate this approach. Indeed, in this case, these new medicines can optimize the therapeutic effect of the drug and in some cases lead to a second pharmacological application of an already well-known drug. The ideal drug candidates for a biopharmaceutical development comprise drugs with poor solubility, permeability and stability when there are administered by another route rather than the intravascular one. As an example, one can mention (i) polypeptide antibiotics (capreomycin, teicoplanin, vancomycin, conjugated ampicillin) which are very active towards Gram-positive and Gram-negative microorganisms, and allow loco-regional or site-specific delivery; (ii) paclitaxel, 5-fluorouracil, tamoxifen or antitumor agents of natural origin for oral therapy in order to improve the bioavailability or to favor a lymphatic uptake; (iii) nucleic acids, guanosine derivatives, adenosine derivatives (ciclopentiladenosine) or in combination with dopamine for nasal delivery in order to target the brain or antigenic peptides in order to induce an immune response.

To overcome these problems, some strategies consist of modifying the chemical structures of the active compounds. Obviously, the chemical modifications will change the physicochemical characteristics of the drugs and will generate new chemical entities.

More recently, alternative strategies have been suggested which consist of development of new products rather than chemical modification of the molecule. Pharmaceutical technology, for example, uses particular excipients that can mask the chemical structure, allowing the drug to pass unmodified for biological availability. Thus, pharmaceutical technology does not generate new drugs but new dosage forms. The development of an already known active compound formulated in an innovative dosage form makes available a new product at lower costs, revaluating active compounds well established in clinical practice. Technology indeed is able to optimize the biological availability of drugs.

To reach these aims, new raw materials, new dosage forms and technologies are required. From a biopharmaceutical point of view, the ideal size of the basic particle has to be in nanometric scale (as described above) but from a pharmaceutical technology point of view, the handling (flow, electrostatic problems...) of bigger particles is easier. Indeed, particles having a very small size *i.e.*, in the nanoscale, show difficulty in free flow and are prone to electrostatic charges on the surfaces. For these reasons, and easy handling the ideal size of the particles has to be in a micrometric range. To satisfy the biopharmaceutical and technological requirements, the best compromise consists of building hybrid micrometric particles which can encapsulate particles of nanometric range.

Hybrid carriers having nano/micro dimensions, also named as Trojan microparticles, have been studied and developed as new drug products capable of carrying and release of active substances with unfavorable biopharmaceutical properties. Indeed, the use of particulate systems can affect their biological availability and is capable of improving drug bioavailability. These particulate vectors can be incorporated into classical dosage forms for different administration routes.

The main goal of these pharmaceutical nano/microtechnologies is based on the discovery of new particulate carriers for drugs exhibiting critical biopharmaceutical or kinetics properties. The use of new raw materials or their combinations coupled to innovative physical structures and new manufacturing procedures allow reaching this aim. Therefore, the Trojan microparticles technologies are focused on the preparation of hybrid particles containing drugs having low solubility, poor permeability and short stability by means of new materials, mechanisms, structures, technologies and manufacturing procedures. These new strategies for the formulation of drugs have lead to the alteration of pharmacokinetics/pharmacodynamics of active substances by modifying their absorption and distribution by means of rate and site controlled delivery.

From a biopharmaceutical point of view, several advantages can be attributed to these particulate technologies, like: (i) reduction of the dose administered, (ii) increasing the bioavailability of poorly soluble and absorbed drugs, (iii) prolonged action of the drug after one administration, (iv) improvement of the therapeutic index and therefore reducing the toxicity, (v) improvement of the compliance of the dose intake by the patients, (vi) possibility of use of non-invasive routes of administration for drugs having unsuitable biopharmaceutical and/or pharmaockinetics properties for administrations alternative to parenteral route and then to favor the pharmaco-economical aspects of certain drugs administrable only by injectable administrations (vii) reduction of the general exposition of the host to the active agent. Indeed, a lot of active compounds, although they are very potent, are not able to reach an effective blood concentration, due to absorption, distribution, half-life or stability problems.

Two classes of raw materials, lipids and polymers, can be used to build these colloidal particles using nano/microtechnologies based techniques. Besides the choice of the nature of the raw materials, the synthesis protocol is also a key factor to be taken into account since this can drastically modify the particle size, surface charge and the physicochemical characteristics (hydrophilic/hydrophobic). Many biocompatible raw materials have been suggested for this purpose which may include phospholipids, medium- and long-chain-triglyceride lipid-based systems, polylactide and copolymers, polysaccharides such as hyaluronic acids, chitosan, alginate *etc*. As mentioned above, these particles can be prepared by various methods using preformed polymers or polymerizing a monomer during the preparation process.

The Trojan microparticles, due to their architecture, can improve the mucosal absorption of drugs either by means of prolonged retention and preferred transport at mucosal site or by protection of carrier substance from degradation. The optimizing of the biopharmaceutical properties of drugs by means of particulate carriers is a real challenge leading to innovation.

Puerarin is an isoflavone isolated from a Chinese herbal medicine, Radix Puerariae Lobatae, and has been reported to have a comprehensive pharmacological action in the treatment of diabetes and cardiovascular diseases. Puerarin is a water-soluble drug which has a short half-life in the body. One way to increase its therapeutic efficacy is to prepare nanoparticles of this active compound and then to microencapsulate using a supercritical CO_2_ process or to use a co-precipitation process using biodegradable polymers to produce a sustained release effect. *In vitro*, Chen *et al.* [10] found that the puerarin nanoparticles were released very quickly through the dialysis bag, and the drug dissolved completely in 6 h. After microencapsulation of puerarin by poly(L-lactide) (PLLA), the puerarin PLLA microparticles showed a burst of release, and about 60.2% of the puerarin was released in the first 4 h; this result demonstrated that this puerarin fraction can be easily removed, which was similar to the results of the encapsulation efficiency measurements. After that, the microparticles released the drug in a sustained process, about 89.6% of the drug was released within 24 h and was finally completely released in 36 h. After incubation in PBS (pH 6.8) for 24 and 48 h, it was seen that after 24 hours the surface became cleaner and some micropores were observed due to the removal of puerarin nanoparticles while after 48 h more micropores were generated by the continuous removal of puerarin nanoparticles.

Thote and Gupta also used supercritical carbon dioxide to microencapsulate nanoparticles of dexamethasone phosphate, a hydrophilic drug, in order to sustain its release [8]. *In vitro* drug release of dexamethasone phosphate nanoparticles encapsulated in PLGA microparticles was compared to the unprocessed drug particles as provided by the supplier also encapsulated in PLGA microspheres. The *in vitro* drug release of the unprocessed drug particles (but microencapsulated) showed an initial burst release of about 5% at time zero, and the entire drug was released within 24 hours. This kinetic release is not advisable for sustained drug release. On the other hand, the *in vitro* drug release of the nanoparticles (also microencapsulated) obtained using supercritical antisolvent technique with enhanced mass transfer (SAS-EM) showed continuous release for 700 hours. The release profile showed almost no burst release, however ~15% was released in the first 60 hours followed by a stationary state of about 200 hours, during which only 22% of the encapsulated drug was released. This was followed with continuous increase in the drug release rate up to 500 hours, during which about 75% of the drug was released followed by continued release of the drug over time. From the sustained drug release profile, the authors clearly inferred that the drug was well dispersed in the polymer matrix, which allowed gradual release of the drug with polymer degradation, as opposed to initial burst release observed in the case where the w/o/o/o phase separation technique was used for encapsulation of dexamethasone phosphate. As the complete process was anhydrous, Thote and Gupta suggested that similar sustained release formulations could also be produced for other hydrophilic drugs.

In order to decrease the initial burst release of low molecular weight drugs from formulations including drug-loaded micro- and nanoparticles, Hasan *et al.* [48] prepared composite microparticles by using w/o/w emulsion process. They encapsulated polymeric nanoparticles into polymeric microparticles by using non-water soluble polymers and appropriate organic solvents. They compared the release kinetics with nanoparticles and classical microparticles prepared by the same method. Poly-ε-caprolactone (PCL) dissolved in methylene chloride was used to make nanoparticles, whereas ethylcellulose and Eudragit RS dissolved in ethyl acetate, a non-solvent of poly-ε-caprolactone, were used for the preparation of microparticles. Ibuprofen and triptorelin acetate were chosen as lipophilic and hydrophilic model drugs, respectively. High entrapment efficiencies were obtained with ibuprofen whereas lower amounts of triptorelin acetate were encapsulated, mainly with formulations prepared with poly-ε-caprolactone and Eudragit RS used alone or blended with ethylcellulose. The authors showed that the burst was significantly lower with composite microparticles and may be explained by the slower diffusion of the drugs through the double polymeric wall formed by the nanoparticle matrix followed by another diffusion step through the microparticle polymeric wall.

The solutions of optimized drug releases given by the technology of Trojan microparticles can be extended to the ocular route. Gómez-Gaete *et al.* [7] combined the therapeutic potential of nanoparticles systems with the ease of manipulation of microparticles by developing Trojan particles. The aim of their new delivery vehicle was to be used for intravitreal administration of dexamethasone (DXM). Indeed, the presence of crystalline drug along with the nanoparticles considerably reduces its ability to load the polymeric matrix. However, Gómez *et al.* demonstrated that 1.3 mg DXM (=1.44 mg dexamethasone acetate (DXA)) could be encapsulated into 100 mg nanoparticles which was about 6 fold more than what could be obtained by using dexamethasone. Initially, they optimized dexamethasone acetate (DXA) encapsulation into biodegradable poly(D,L-lactide-co-glycolide) (PLGA) nanoparticles, then Trojan particles were formulated by spray drying 1,2-Dipalmitoyl-sn-Glycero-3-Phosphocholine (DPPC), hyaluronic acid (HA) and different concentrations of nanoparticle suspensions. The effects of nanoparticles concentration on the physical characteristics of Trojan particles as well as the effect of the spray drying process on nanoparticles size were investigated. Finally, DXA *in vitro* release from nanoparticles and Trojan particles was evaluated under sink condition. Although a burst effect can be observed with both systems, the extent of the burst was lower for Trojan particles. SEM and confocal microscopy confirmed that most of Trojan particles were spherical, hollow and possessed an irregular surface due to the presence of nanoparticles. The authors observed that neither Trojan particle tap density nor size distribution was significantly modified as a function of nanoparticles concentration. They mean size distribution of nanoparticles was also found to increase significantly after spray drying. Finally, based on *in vitro* release of DXA they showed that the excipient matrix provided protection to encapsulated nanoparticles by slowing down drug release. Gómez *et al.* concluded that even if the active principle was released rather quickly as compared with other implants, the *in situ* release of drug loaded nanoparticles should favor their internalization within retinal pigment epithelial cells and might therefore increase the drug efficacy for treating retinal affections.

New transmucosal administration routes, like the pulmonary, could also be exploited for systemic absorption of drugs. Edwards *et al.* [51] were the first to show that very light particles (*ρ* <~ 0.4 g/cm^3^) with d > 5 μm can be deposited in the lungs. As a consequence of their large size and low mass density, porous particles can aerosolize from a DPI more efficiently than smaller nonporous particles, resulting in higher respirable fractions of inhaled therapeutics. Also by virtue of their size, large particles can avoid phagocytic clearance from the lungs until the particles have delivered their therapeutic dose; this attribute can be particularly useful for controlled-release inhalation therapies. To optimize physiologically the kinetic release of the drug incorporated into these Large Porous Particles (LPPs) and to allow producing them easily, Tsapis *et al.* [22] combined the drug release and delivery potential of nanoparticle systems with the ease of flow, processing, and aerosolization potential of Large Porous Particle (LPP) systems by spray drying solutions of polymeric and nonpolymeric NPs into extremely thin-walled macroscale structures. These hybrid LPPs exhibited better flow and aerosolization properties than the NPs; yet, unlike the LPPs, which dissolved in physiological conditions to produce molecular constituents, the hybrid LPPs dissolved to produce NPs, with the drug release and delivery advantages associated with NP delivery systems. Formation of the large porous NP (LPNP) aggregates occurred via a spray-drying process that ensured that the drying time of the sprayed droplet was sufficiently shorter than the characteristic time for redistribution of NPs by diffusion within the drying droplet, implying a local Peclet number much greater than unity. Additional control over LPNPs physical characteristics were achieved by adding other components to the spray-dried solutions, including sugars, lipids, polymers, and proteins. The ability to produce LPNPs appeared to be largely independent of molecular component type as well as the size or chemical nature of the NPs. Hadinoto *et al.* [42,52] developed similar drug delivery systems based on large hollow nanoparticulate aggregates as therapeutic carrier particles in dry powder inhaler delivery of nanoparticulate drugs. The large hollow carrier particles were manufactured by spray drying of nanoparticulate suspensions of biocompatible acrylic polymer with loaded drugs. The size and concentration of the nanoparticles, as well as the phospholipids inclusion, have been known to influence the resulting morphology (*i.e.*, size and degree of hollowness) of the spray-dried carrier particles. The effects of the resulting morphology of the carrier particles on the drug release rate were therefore investigated by varying the above three variables. To study these different factors, the authors used aspirin and salbutamol sulfate since these two model drugs have a varying degree of solubility in water. The results indicated that the drug release rate is mostly governed by the degree of hollowness of the carrier particles, and also to a lesser extent by the nanoparticles size, where variation in the drug loading capacity of nanoparticles was observed for different particle size.

Design of appropriate inhaled carriers with adequate aerodynamic properties, drug release, biodegradation and evasion of macrophage uptake is a major challenge for controlled release pulmonary drug delivery [53,54]. In order to reach this aim, Al-Qadi *et al.* [55] and El-Sherbiny *et al.* [56] prepared hybrid PEG graft copolymerized onto N-phthaloyl chitosan and chitosan/hyaluronic acid nanoparticles, by ionotropic gelation, and microencapsulated them respectively in sodium alginate and mannitol microspheres, resulting in a dry powder that showed adequate aerodynamic properties for deep pulmonary deposition. Following the encapsulation process, structural analysis of the dry powder was analyzed by confocal laser scanning microscopy, which elucidated that the nanoparticles were homogeneously distributed within the mannitol microsphere. The evidence that nanoparticles were completely encapsulated within the carrier by means of the spray drying process was achieved by application of the sensitive surface analysis techniques, X-ray photoelectron spectroscopy and in combination with time-of-flight secondary ion mass spectroscopy. These outcomes confirmed the success of nanoparticles microencapsulation by spray drying and the authors concluded that the developed delivery system holds great potential for lung delivery of macromolecules. Grenha *et al.* [43,57] concluded similarly by studying chitosan nanoparticle-loaded mannitol microspheres. The mannitol microspheres contained chitosan/tripolyphosphate nanoparticles and were also prepared by spray-drying. These microspheres were proposed as valuable candidates to transport therapeutic protein-loaded nanoparticles to the lungs owing to their favorable aerodynamic properties. Similar formulations were developed by other researchers in order to open the way for new drug-targeting strategies using nanoparticles for proteins [58] and pulmonary delivery of drugs and diagnostics. Indeed, some of them [59] developed a platform for aerosol delivery of nanoparticles. In this case, lactose was used as the excipient and spray-dried with two different types of nanoparticles: gelatin and polybutylcyanoacrylate nanoparticles. The results of these studies showed that some carrier particles were hollow while others had a continuous matrix. Gelatin nanoparticles were incorporated throughout the matrix and sometimes accumulated at one end of the lactose. Polycyanoacrylate nanoparticles mostly clustered in different spots within the lactose carriers. The mean sizes of both nanoparticle types were characterized at two different times: one just before they were spray-dried and other after they were redissolved from the spray-dried powders. Both nanoparticle types remained in the nano-range size after spray-drying. The mean nanoparticle sizes were increased by approximately 30% after spray-drying, though this increase was statistically significant only for the gelatin nanoparticles. Dispersion of the powder with an in-house passive dry powder inhaler and subsequent cascade impaction measurements showed that incorporation of the nanoparticles did not affect the fine particle fraction (FPF) or mass median aerodynamic diameter (MMAD) of the powders. FPF was approximately 40% while MMAD was 3.0 ± 0.2 μm, indicating these formulations yielded aerosols of a suitable particle size for efficient lung delivery of nanoparticles. As for the other delivery systems intended for this administration route, the researchers demonstrated that nanoparticles can be delivered to the lungs via carrier particles that dissolve after coming in contact with the aqueous environment of the lung epithelium.

Besides the specific uses of Trojan microspheres for pulmonary delivery, the particles have also found applications for oral administration, to reach desirable sustained release profiles. El-Sherbiny *et al.* [60] developed a series of nano/micro hydrogel matrices for oral delivery of silymarin. This drug was chosen as a model of hydrophobic natural therapeutics. Constituted of sodium alginate-based pH responsive hydrogel microspheres encapsulating poly(D,L-lactic-co-glycolic acid) (PLGA) nanoparticles (NPs), this new design of particles allowed enhancing the dissolution and oral bioavailability of this model drug via formulation in polymeric matrices. From these studies, the authors showed that alginate-based hydrogel microparticles incorporating silymarin-loaded PLGA NPs are pH-sensitive. Also, the obtained data revealed a considerable effect of the alginate content and the drying method onto the characteristics of the prepared alginate-PLGA particles.

In the field of gene delivery by oral route, Bhavsar *et al.* [49,61] investigated the development and the evaluation of a novel nanoparticles-in-microsphere oral system (NiMOS) for gene delivery and transfection in specific regions of the gastrointestinal (GI) tract. To achieve this, plasmid DNA, encoding either for β-galactosidase (CMV-βgal) or enhanced green fluorescent protein (EFGP-N1), was encapsulated in type B gelatin nanoparticles. NiMOS were prepared by further protecting the DNA-loaded nanoparticles in a poly(epsilon-caprolactone) (PCL) matrix to form microspheres of less than 5.0 μm in diameter. In order to evaluate the biodistribution following oral administration, radiolabeled (^111^In-labeled) gelatin nanoparticles and NiMOS were administered orally to fasted Wistar rats. The results of biodistribution studies showed that, while gelatin nanoparticles traversed through the GI tract rather quickly with more than 85% of the administered dose per gram localizing in the large intestine within the first hour, NiMOS resided in the stomach and small intestine for relatively longer duration. Following oral administration of CMV-βgal or EFGP-N1 plasmid DNA at 100 μg dose in the control and test formulations, the qualitative results presented in this study provide the proof-of-concept for the transfection capability of NiMOS upon oral administration. After 5 days post-administration, the authors observed transgene expression in the small and large intestine of rats. Based on these preliminary results, NiMOS showed significant potential as novel gene delivery vehicle for therapeutic and vaccination purposes.

Several other peculiar applications have been investigated for the Trojan microparticles. These ones comprise the possibility to incorporate iron oxide. In this context, superparamagnetic iron oxide nanoparticles embedded chitosan microspheres were developed for magnetic resonance (MR)-traceable embolotherapy with high resolution and sensitivity through magnetic resonance imaging (MRI) [62]. Superparamagnetic iron oxide nanoparticles loaded chitosan microspheres were prepared by emulsion and cross-linking technique and 100–200 μm sized spherical microsparticles were obtained. Within 30 days, about 60% of the incorporated superparamagnetic iron oxide nanoparticles were released from low cross-linked microspheres, whereas only about 40% of superparamagnetic iron oxide nanoparticles were released from highly cross-linked microspheres. Highly cross-linked microspheres were more efficient for lower degree of swelling leading to secure entrapment of superparamagnetic iron oxide nanoparticles in matrix.

Drug-loaded microspheres using the proof-of-concept of Trojan particles, a kind of target-orientation drug, were also investigated by several researchers. Drug delivery systems constituted of a magnetic polymeric carrier, composed of biodegradable poly(D,L-lactide) microspheres, maghemite nanoparticles and anti-cancer drug (paclitaxel) was successfully prepared in dichloromethane using highspeed homogenization [63]. Maghemite nanoparticles were well dispersed in poly(D,L-lactide) matrix. The carrier was magnetically responsive and release of loaded paclitaxel was enhanced by applying an oscillating magnetic field. The thermal energy generated by maghemite nanoparticles due to magnetic hysteresis loss was very low and had a negligible effect in influencing the release behavior. The authors showed that alternating movement of the nanoparticles, stimulated by magnetic force, resulting in deterioration of the mechanical properties of polymer matrix was likely to be the main reason for the enhancement in drug release. Similar systems containing paclitaxel or interferon alpha-2b were respectively formulated by Cui *et al.* [64] and Zhou *et al.* [65].

In an anecdotic way, one can also mention the use of hybrid particles for the enzyme immobilization. Sadjadi *et al.* [66] investigated the assembly of the silver nanoparticles on the surface of the amine-functionalized zeolite microspheres or formation of zeolitesilver nanoparticle “core-shell” structure and thereafter, using this nanosystem in immobilization of fungal protease. The assembly of silver nanoparticles on zeolite surface occurs through the amine groups present in 3-aminopropyltrimethoxysilane. The fungal proteases bound to the massive “core-shell” structures can be easily separated from the reaction medium by mild centrifugation and exhibited excellent reuse characteristics. The biocatalytic activity of fungal protease in the bioconjugate was marginally enhanced relative to the free enzyme in solution.

Using the technology of the encapsulation of nanoparticles into microspheres, Lee *et al.* [67] reported the antibacterial properties of magnetically directed microparticles containing Ag nanoparticle loaded multilayers. Original development of Trojan microspheres was realized in tissue-regeneration matrix and drug delivery systems. Park *et al.* [68] applied these biomaterials as novel cell supporting matrix for stem cell delivery. They devised a novel method for the fabrication of nanostructured 3D scaffolds. The growth factor loaded heparin/poly(L-lysine) nanoparticles were physically attached on the positively charged surface of PLGA microspheres precoated with low molecular weight poly(ethyleneimmine) via a layer-by-layer system. Growth factor loaded heparin/poly(L-lysine) nanoparticles, which were simply produced as polyion complex micelles with diameters of 50–150 nm, were fabricated in the first step. Microsphere matrix (size, 20~80 nm) containing TGF-β 3 showed a significantly higher number of specific lacunae phenotypes at the end of the 4 week study *in vitro* culture of mesenchymal stem cells. Thus, the researchers concluded that growth factor delivery of polylactide-co-glycolide microsphere can be used to engineer synthetic extracellular matrix. This polymeric microsphere matrix containing TGF-*β* 3 showed promise as coatings for implantable biomedical devices to improve biocompatibility and ensure *in vivo* performance.

## 4. Conclusion

As demonstrated above, the hybrid particles present a lot of pharmaceutical interests since these technologies are able to envisage the desirable pharmacokinetic for a drug. They should contribute towards improvement of targeting properties of a drug; decrease the dosage amount to be administered to a patient and to improve the comfort of patients by minimizing side effects. From a toxicological point of view, since the greater surface area per mass compared with larger-sized particles of the same chemistry renders nanosized particles more active biologically, these Trojan particles have to be evaluated toxicologically as microspheres and nanoparticles. As nanoparticles, their toxicological evaluation includes a potential for inflammatory and pro-oxidant, but also antioxidant activity, which can explain early findings showing mixed results in terms of toxicity of nanosized particles [69]. Evidence of mitochondrial distribution and oxidative stress response after nanosized particle endocytosis and their possible interactions with subcellular structures have also to be studied. Additional considerations for assessing safety of engineered nanosized particles include also careful selections of appropriate and relevant doses/concentrations, the likelihood of increased effects in a compromised organism, and also the benefits of possible desirable effects.
